# Manual therapeutic plasma exchange for treatment of a dog with suspected acute canine polyradiculoneuritis

**DOI:** 10.1186/s13028-023-00675-0

**Published:** 2023-03-27

**Authors:** Adriana Czerwik, Tereza Jarešová, Agnieszka Olszewska, Daniela Farke, Martin Jürgen Schmidt, Hendrik Lehmann

**Affiliations:** 1 Department of Veterinary Clinical Sciences, Neuroradiology and Clinical Neurology, Small Animal Clinic, Justus-Liebig-University, Frankfurter Str.114, 35392 Neurosurgery, Giessen, Germany; 2 Department of Veterinary Clinical Sciences, Internal Medicine, Small Animal Clinic, Justus- Liebig-University, Frankfurter Str.114, 35392 Giessen, Germany; 3 Department of Emergency and Critical Care Service, Vetklinikum LS, Laxenburger Str. 252a, 1230, Vienna, Austria

**Keywords:** Canine, Guillain-Barré syndrome, Neuromuscular disease, Therapeutic plasma exchange

## Abstract

**Background:**

Acute canine polyradiculoneuritis is one of the most common polyneuropathies occurring in dogs. The disease is very similar to the Guillain–Barré syndrome in humans. In veterinary medicine, there is no established treatment for this disease, while in human medicine, therapeutic plasma exchange and intravenous immunoglobulin administration are two main immunotherapy treatments of this syndrome.

**Case presentation:**

A 12-year-old male Jack Russel Terrier was presented with a history of acute weakness of the pelvic limbs progressing to flaccid tetraplegia with respiratory compromise. Complete diagnostic workup was performed including blood work, diagnostic imaging (radiographs of the thorax as well as ultrasound of the abdomen) and echocardiography. Based on the clinical course, neurological localisation and the results of electrodiagnostic examination acute canine polyradiculoneuritis was suspected. During the hospitalization, the dog deteriorated and was admitted to the intensive care unit for respiratory support via tracheostomy tube. In addition to symptomatic treatment, immunotherapy via single treatment of manual therapeutic plasma exchange was administered. This procedure was safe, and the dog showed improvement of clinical signs 3 days after therapy was initiated, as well as improvement of neurological signs (from grade 4 tetraplegia to grade 3) within 5 days. However, the dog was euthanized 3 weeks later due to complications related to the tracheostomy.

**Conclusions:**

This is the first case report of a manual therapeutic plasma exchange in a dog with suspected acute canine polyradiculoneuritis suggesting that this method is safe and well tolerated in dogs with this disease. It may be a reasonable adjunctive treatment to supportive therapy in severe cases.

**Supplementary Information:**

The online version contains supplementary material available at 10.1186/s13028-023-00675-0.

## Background

Acute canine polyradiculoneuritis (ACP) is one of the most common polyneuropathies occurring in dogs [1]. The disease is very similar to Guillain–Barré Syndrome (GBS) in humans, caused by the immune-system inflicted damage of the peripheral nervous system. In veterinary literature, the disorder is also known as coonhound paralysis or idiopathic polyradiculoneuritis [1, 2]. Although the pathogenesis of ACP is uncertain, an autoimmune process affecting both axons and myelin of the ventral nerve roots is suspected. The proposed pathogenesis includes molecular mimicry or the production of autoantibodies against axolemmal components such a anti-ganglioside antibodies (Abs)[1, 3]. The first clinical signs in affected dogs usually develop in the pelvic limbs and eventually progress to the thoracic limbs, causing generalized lower motor neuron paresis/plegia. Most affected patients develop non-ambulatory tetraparesis/tetraplegia within 10 days of the onset of clinical signs [4]. The prognosis for full recovery is usually favourable, if adequate supportive treatment is applied, except in cases with life-threatening respiratory impairment [5], where full recovery is prolonged, taking several weeks to several months or it may even lead to death. There is no known disease-specific therapy described in veterinary medicine. Hence, treatment options for ACP are warranted. A recent study showed that dogs suffering from ACP treated with intravenous immunoglobulin (IVIg) [5] recovered faster compared with dogs without immunotherapy. Dogs suffering from myasthenia gravis, another neuromuscular, autoimmune disease treated with therapeutic plasma exchange (TPE), recovered faster compared with dogs without immunotherapy [6]. In human medicine, plasma exchange as well as IVIg have become the gold standard treatment for GBS [7].

This case report describes the clinical and neurological presentation, electrodiagnostic findings and manual therapeutic plasma exchange treatment in a small breed dog with suspected ACP.

## Case presentation

A 12-year-old male Jack Russel terrier was referred to the Department of Veterinary Clinical Sciences in the Small Animal Clinic of the Justus-Liebig-University Giessen, Germany with a history of symmetrical weakness which began in the hindlimbs, evolved to the forelimbs, and progressed to a non-ambulatory flaccid tetraplegia within 4 days. The medical history revealed no information about possible trauma, concomitant illnesses, or exposure to neurotoxins. Apart from a left apical systolic heart murmur grade 5, there were no abnormalities present on the physical examination. The neurological examination revealed lack of postural reactions and absent spinal reflexes (flaccid tetraplegia). On examination of the withdrawal reflex, the dog showed hyperesthesia. Neck movements were weak, and the dog could not lift his head. The perineal reflex and cranial nerves were intact. The patient maintained the ability to move its tail and to urinate and defecate. Moderate, generalized muscle atrophy was visible. The clinical signs were localized to the peripheral nervous system involving the motor nerve roots.

The neurological examination was followed by laboratory analysis including complete blood count, serum biochemical profile (glucose, total protein, albumin, urea nitrogen, creatinine, total bilirubin, alanine and aspartate transferase, alkaline phosphatase, creatine kinase, lipase, cholesterol, Na^+^, PO_4_^−^, iCa^+^, K^+^, iMg^+^ and c-reactive-protein), thyroid hormones (TSH, T4) and antibody levels against *Neospora caninum* and *Toxoplasma gondii*. The dog underwent radiographic examination of the thorax as well as ultrasound examination of the abdomen and echocardiography.

The CBC, serum chemical and electrolyte analyses were within reference ranges, except for a decreased T4 level (0.7 µg/dl with the reference 1.5-4 µg/dl) with a normal TSH level (0.09ng/ml with the reference up to 0,30bg/ml), which was attributed to the euthyroid sick syndrome. The antibody results for *N. caninum* and *T. gondii* were negative with IgG titer 1:80, IgM titer < 1:40 (reference < 1:40) and IgG titer < 1:64, IgM titer < 1:16 (reference < 1:64) respectively. The results of the radiological examinations (radiographs as well as ultrasound) showed no clinically relevant abnormalities. The cardiological examination revealed a mitral valve insufficiency with a moderate enlargement of the left atrium, indicating a stage B2 myxomatous mitral valve degeneration (MMVD). All results from imaging diagnostics including echocardiography were reviewed by board certified radiologist and cardiologist.

**Fig. 1:**
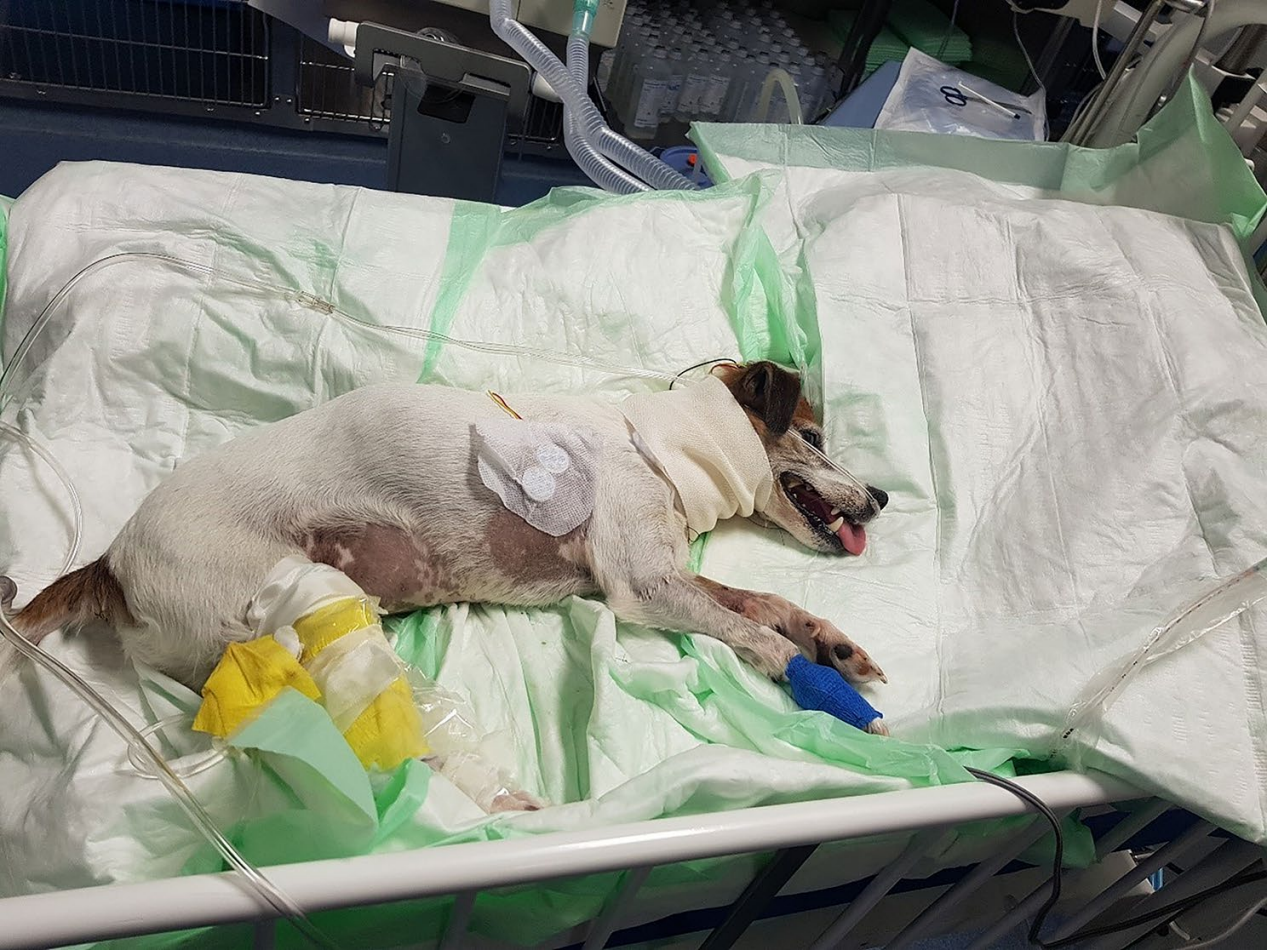
The dog during a stay in the intensive care unit

The dog was admitted to the hospital for supportive care and began to show signs of mild dyspnoea, loss of appetite and apathy on the second day of hospitalization. At this stage, respiratory movements were shallow and primarily abdominal. On the following day, the dog was hypoventilating and was assigned to the intensive care unit for ventilatory support (mechanical ventilation) (Fig. 1). Ventilatory support was achieved using pressure-controlled ventilation allowing the patient to trigger each respiratory cycle with use of the respirator software (Servo-S, Maquet Getinge Group, Rastatt, Germany). A tracheostomy tube was placed to improve care and regulate the dog’s condition with minimal sedation. The dog’s condition did not change during the first three days of intensive care.

Seven days after clinical onset, electrophysiological tests were performed under general anaesthesia with the dog in lateral recumbency, using a Nicolet VikingQuest System (Electrodiagnostic Software, USA). The electromyographic examination (EMG) revealed moderate insertional activity and fibrillation potentials in the quadriceps, cranial tibial and plantar interosseus muscles. Motor nerve conduction velocity (MNCV) of the tibial nerve was measured following proximal (trochanter), middle (caudal stifle) and distal (hock) stimulation using monopolar needle electrodes. Compound action potentials (CMAPs) were recorded from the plantar interosseus muscle. The distal latency and amplitude of the CAMPs were measured. MNCV was calculated from the trochanter to the stifle and the stifle to the hock segment of the tibial nerve and revealed a moderate reduction in the MNCV of the tibial nerve (proximal 26 m/sec, distal 63 m/sec, with the reference 60–70 m/sec and 70–80 m/sec respectively) [8]. The tibial residual latency was normal (1.72 m/sec, with the reference 1.6-2 m/sec) [8]. F-waves were measured using the same electrode configuration and were recorded at the plantar interossei muscles. An increased F-wave latency of 16.4 m/sec was detected (minimal expected F-wave latency was 13 m/sec). The calculated F-ratio was higher than the reference values for the stifle (1.67 vs. 0.8), which indicated more severe involvement of the proximal part of the nerve. Neuromuscular transmission was assessed using repetitive nerve stimulation (RNS) at the hock at 2 and 3 Hz recorded at the plantar interosseous muscles, to exclude a neuromuscular junction disease. RNS was normal with no decrement.

**Fig. 2:**
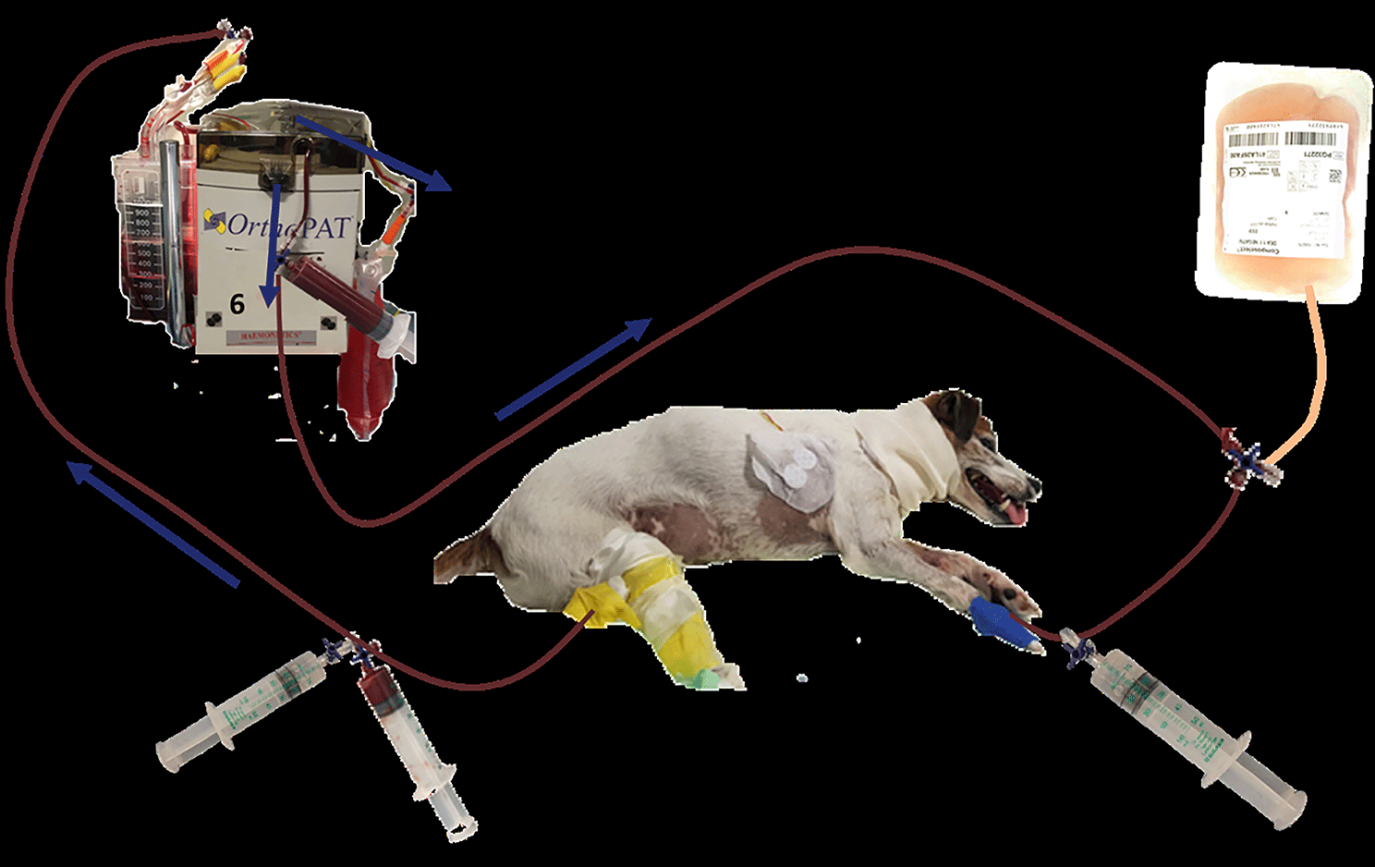
Scheme of the approach to the plasma exchange (1) Aspiration of dog’s blood into a syringe and injection of this blood into the autotransfusion system; (2) Anticoagulation of the aspirated blood with sodium citrate; (3) separation of dog’s blood into plasma and red blood cells in the autotransfusion system; (4) collecting bag for dog’s plasma; (5) donor plasma; (6) aspiration of the erythrocyte concentrate from the autotransfusion system in exange with aspiration of donor plasma, injection of the erythrocytes or plasma into the dog; (7) substitution of calcium as required

After three day of ventilatory support, single manual TPE was performed. A commercial autotransfusion set used for orthopedic procedures and described elsewhere [9] was applied to the existing peripheral central venous catheter (3 F 200 mm Careflow One Lumen catheter, Merit medical Ireland Ldt, Galway, Ireland) in the left cephalic vein. A second peripheral central venous catheter was placed into the right saphenous vein. Although use of some commercially available TPE systems is described in small sized dogs with corresponding blood priming related to the large extracorporal needed [10–12], those systems were not available in the presented case. A therapeutic plasma exchange system was built between the two catheters consisting of a set of infusion lines, a modified autotransfusion system (Orthopat, Haemonetics inc, Boston, MA, USA) and a bag of donor plasma. Fifty ml of blood was manually drawn and anticoagulated with sodium citrate (sodium citrate 3.13%, Eifelfango) taken from the peripheral central venous catheter and transferred to the reservoir of the system (Fig. 2). The blood was aspirated and centrifuged, the plasma separated, and the blood additionally washed with 0.9% saline (isotonic sodium chloride ad us vet., B. Braun Vet Care) according to the manufacturer’s instructions. Following the centrifugation process, the blood was automatically transferred as an erythrocyte concentrate into a sterile 50 mL syringe. Subsequently, these washed cells were administered to the dog through the other catheter with a corresponding amount of a donor plasma.

This cycle was repeated until as much as 1.16 of total plasma volume was replaced. The amount of exchanged plasma volume in our case was comparable to exchanged plasma volume in another study [13], which used 1.0 times total plasma volume. This number was extrapoliated from human medicine, where usually in a single TPE procedure 1–1.5 of total plasma volume is replaced [14]. Exchange of larger volumes is not reported to be more efficient and the risk of side-effects is increased [15].

The total plasma volume was assumed according to the following formula: 0.09 x BW [4.7] x (1-haematocrit [0.4]) and calculated to 250 mL. The exchanged plasma volume was 290 mL and the procedure of the exchange took approximately 3 h. Because of the risk of an immunologic reaction and haemodynamic complications, the dog was connected to a medical monitor (Carescape B650, GE HealthCare, Chicago, IL, USA) and the vital functions (ECG, respiratory rate and pattern, body temperature, NIBP, SpO_2_) were continuously monitored during the whole procedure. Due to the administration of a large volume of donor plasma containing calcium citrate to prevent blood-clotting, there was a risk of developing a clinically relevant hypocalcaemia. Hence, blood gas analyses were repeatedly performed (after exchange of half of the calculated plasma volume, immediately after the procedure and 10 h thereafter). Calcium deficiency was substituted with calcium gluconate(calcium gluconate B. Braun 10%) as required (Additional file 1). Except gradual decrease of ionized calcium (1.16 mmol/L before TPE to 0.708 mmol/L) during the procedure, no other complications were observed. Clinical signs related to hypocalcaemia such as cardiac arrhythmias or tetanic muscle contractions were not observed.

Over the following two days, the clinical signs were slowly improving, and the dog gradually required less mechanical support compared to previous days. On day 3 after TPE, the dog was completely weaned off the ventilator and was able to breath independently without signs of dyspnoea or hypoventilation. An increase of alertness and responsiveness to its environment was also noticed and the dog´s appetite improved. The tracheal tube was removed on day 3 post TPE. On day 5, the dog returned to the neurological department and showed a mild neurological improvement. The dog was able to hold its head when placed in sternal recumbency while eating and showed voluntary movement of the limbs. The dog was discharged the following day with recommended intensive and regular physiotherapy as well as post-tracheostomy wound care (bandage change). No pharmacological therapy was needed other than pimobendane for the MMVD Stage B2.

One week following discharge, the dog returned to the hospital for a follow-up examination. The owners reported that the dog had been doing well at home and tried to crawl. Apart from generalized, advanced muscle atrophy and persistent heart murmur at the level of the mitral valve, the general physical examination was unremarkable. During the neurological examination the dog was able to lift its head and to keep it in an upward position without assistance. The dog showed voluntary movement in all limbs but was not able to ambulate. The muscle tone was improved but remained mildly decreased. Postural reactions were decreased and the absence of the patellar, tibialis cranialis and extensor carpi radialis reflexes remained. The withdrawal reflex was reduced in the pelvic limbs and absent in the thoracic limbs. The dog maintained the ability to move its tail and to urinate and defecate.

**Fig. 3:**
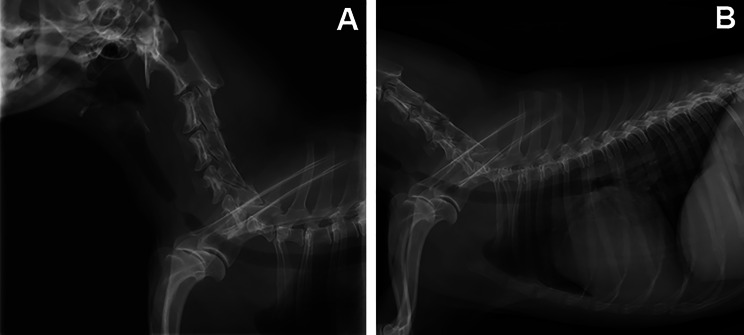
Radiographic features of the trachea (A: neck) and (B: chest) in laterolateral view, showing its advanced stenosis at the height of the entrance in the chest

The dog returned to the emergency service 5 days after the first clinical check-up with severe inspiratory and expiratory dyspnoea and cyanosis. Radiographs of the neck and chest revealed an advanced soft tissue tracheal stenosis in the area where the tracheostomy was initially performed (Fig. 3). The prognosis was guarded, the only possible treatment was insertion of the tracheal stent.

Due to the high cost of further treatment, the owners decided to have the dog euthanized and the owners declined further necropsy .

## Discussion and conclusions

Acute canine polyradiculoneuritis is an acquired peripheral neuropathy primarily involving the ventral nerve roots characterized by the rapid development of a non-ambulatory, lower motor neuron tetraparesis/tetraplegia [4]. GBS is considered the human counterpart of ACP. Its exact pathogenesis remains unknown. Potential triggers, which activate the immune system, are thought to include exposure to bacteria such as *Campylobacter jejuni*, *Mycoplasma pneumonia*, and *Haemophilus influenzae* and viruses such as cytomegalovirus and Epstein-Barr virus, acting as a kind of molecular mimicry [7]. Autoimmune factors such as antibodies are thought to cause the disease. Therefore, TPE may be used to treat GBS in humans. TPE was first reported in the treatment of GBS between 1978 and 1981 and it was the first GBS treatment proven to be superior to supportive treatment alone [16, 17]. The technique works by separating plasma from cells using membrane filtration or centrifugation. While plasma is removed from the patient, cells are reinfused. TPE aims to remove the antibodies from the blood stream and replace them by plasma products (plasma exchange) or a combination of crystalloids, fresh frozen plasma and colloids (synthetic or albumin). IVIg was shown to be equally effective [18–21] and now both techniques are commonly used as first-line treatment for GBS. However, there are reports suggesting that patients with severe GBS may benefit from TPE after immunoglobulin treatment in refractory cases [16, 17]. TPE should be considered in GBS cases with early axonal involvement and in the recurrent or familial GBS forms. Humans treated with TPE or IVIg had significantly faster motor function recovery and required less frequent ventilatory support compared to untreated patients [7, 16].

It is worth mentioning that in GBS, anti-ganglioside Abs are considered important inflammatory mediators. Serum anti-ganglioside Abs were previously reported in ACP in dogs suggesting that the disorder is the canine equivalent to GBS in humans and may share a similar pathophysiology [2]. However, this measurement is not commercially available, yet.

The diagnosis of ACP in dogs is based particularly on history provided by the owners and characteristic clinical signs. Additionally, routine blood tests including thyroid hormones (TSH, T4), the antibody levels against *N. caninum* and *T. gondii* as well as thoracic radiographs and abdominal ultrasound should be performed to rule out metabolic and infectious as well as para-neoplastic causes, respectively [4]. An electrophysiological examination should be carried out in dogs suspected of ACP as some findings are reliable indicators of the disease. EMG typically reveals denervation potentials such as fibrillations and positive sharp waves in the affected muscles, however electromyographic abnormalities may not be detectable for five to seven days following denervation [1]. MNC velocity values remain normal or mildly decreased. The minimum F-wave latencies are prolonged, and the F-ratios are increased. The RNS with a loss of amplitude within 10% is considered physiological, pointing to no disturbance in neuromuscular transmission. Such findings indicate a motor axonopathy and, with concurrent prolonged F-wave latencies as well as F-ratios, indicate more severe involvement of the proximal portions of motor nerves, ventral roots, or both [4, 22]. The results of the electrodiagnostic examination of the presented dog corresponded to the described results suggesting a simultaneous impairment of ventral roots. Neuromuscular junctions were intact excluding a myasthenic syndrome or botulism. The histopathological results of a nerve biopsy can also support the diagnosis [1]. They may contain perivascular and interstitial lymphocytic infiltrations, demyelination, and axonal necrosis of motor nerves, but are usually nonspecific because the primary non-suppurative inflammation is most prevalent in the ventral roots and spares the more peripheral parts of the nerves [2, 23]. Therefore, biopsies were not taken in the described case.

It is worth mentioning that in GBS, anti-ganglioside Abs are considered important inflammatory mediators. Serum anti-ganglioside Abs were previously reported in ACP in dogs suggesting that the disorder is the canine equivalent to GBS in humans and may share a similar pathophysiology [2]. However, this measurement is not commercially available, yet.

In veterinary medicine, there is no specific treatment for ACP. The reported treatment of ACP is limited to physical rehabilitation, supportive care, and proper nutrition. Despite it being an immune-mediated disorder, treatment with corticosteroids does not improve the condition of the dogs [1, 5]. Similarly, steroids are not effective for the treatment of GBS and prolonged corticosteroid therapy may slow recovery [7]. Dogs receiving supportive treatment recover within 4–8 weeks [5]. However, prolonged recovery of up to 3 months or lack of improvement are also reported [1, 4, 5]. Although TPE has been used for immune-mediated diseases (such as immune-mediated haemolytic anaemia = AIHA and thrombocytopenia = ITP) other than myasthenia gravis [6, 24, 25], there is no information of its use as a treatment for dogs with ACP. In dogs with ACP, one study reports a clear trend towards faster recovery in dogs treated with IVIg (a median of 27.5 days, range 15–127 days) in comparison to the control group (a median 75.5 days, range 5–220 days). These results indicate a possible beneficial use of IVIg treatment in dogs with ACP [5]. In another study with immune-mediated myasthenia gravis, three dogs were treated with TPE [6]. TPE was well tolerated in those dogs and was regarded as a safe adjunctive therapy in patients that did not respond to conventional medical treatment with anticholinesterase and immunosuppressive drugs [6]. In the presented case, the dog developed clinical complications resulting from paralysis of the respiratory muscles. We decided to treat the dog with TPE which, as mentioned above, is reported to be superior to IVIg in humans with severe or recurrent forms of GBS.

There are three possible systems or principles which can be used for the TPE procedure. According to literature, membrane based TPE using commercial extracorporeal sets consisting of a membrane filter that separates the plasma and blood cells while adding donor plasma before retransfusing the blood, is the most used system [24]. Alternatively, centrifugation-based systems with single-use sets are available, separating the blood cells from the plasma via centrifugation and adding donor plasma before retransfusion. There have been reports of manual TPE procedures [26]. As previously mentioned, we used a modified commercial autotransfusion set common in orthopaedic procedures, via the existing peripheral central venous catheter.

The dog in this report showed daily improvement of the clinical status after treatment with TPE. After the first day, the dog showed a more stable breathing pattern and was able to breath independently on the third day. There was an improvement of the dog´s general condition, activity, and appetite. On the fifth day, the dog returned to the neurological department from the intensive care unit with a mild improvement of the neurological status showing voluntary movements of the pelvic limbs without ambulation and ability to breath unassisted. On the first control, 11 days after treatment with TPE, the neurological condition of the dog, including muscle tone as well as spinal reflexes, improved, and the owner reported daily improvement of his status after the discharge. This indicated that the dog tolerated the TPE well, and there were no complications following the therapy.

In humans, approximately one third of patients with GBS develop respiratory compromise requiring ICU admission. About half of them need to be intubated and mechanically ventilated (MV). The median duration of MV in humans is 21–27 days, although a subset of them requires prolonged ventilation over several weeks till months [27–29]. There is only limited information about the median time of MV required in neurological disease in veterinary literature. However, one study focused on the outcome of peripheral neurological diseases that required MV where median time of ventilation was 109 h and 4 dogs with ACP were included requiring 55 to 253 h of MV [30]. The clinical condition of the dog started to improve after the first day of TPE which resulted in an early discontinuation (after 3 days) of MV. We speculate that manual TPE therapy might have contributed to the rapid clinical improvement and resulted in an earlier discontinuation of MV.

The death of the dog was not related to the primary disease and the treatment with TPE. The dog showed progressive neurological improvement and no signs of respiratory disease. Development of respiratory distress was a complication to tracheostomy, which was diagnosed more than 2 weeks after completed treatment with TPE. Tracheostomy is a commonly performed intensive care unit procedure. Traditionally, its use was restricted to the emergency management of upper airway obstruction but has recently been also indicated in prolonged mechanical ventilation [29]. The advantages of tracheostomy include the animal’s comfort, safety, no need to keep the animal under general anaesthesia as well as better oral and airway care. Moreover, animals with tracheostomy may have shorter intensive care unit stays and shorter periods of mechanical ventilation and hospital stays as this procedure facilitates care outside the ICU [29]. In our case, the dog developed life-threatening respiratory paralysis due to ACP, which required MV. We decided to perform tracheostomy because of the indications described above and to enable the regular evaluation of the dog’s clinical and neurological condition with minimal use of sedation. During the hospitalization, there were no observed complications following tracheostomy. During the first control, the wound was healing well, and the dog had no signs of respiratory compromise. Five days after the first control, the dog returned to the clinic during emergency hours because of acute, severe inspiratory and expiratory dyspnoea and cyanosis. A soft tissue tracheal stenosis at the level of the previous tracheostomy site was diagnosed on radiographs. In humans, the most significant long-term complication of tracheostomy is tracheal stenosis, which affects 11-17.8% of patients. It is thought that increased pressure against the tracheal rings causes cartilage erosion and results in activation of inflammatory processes, producing a generous stoma [31, 32]. Complications in the form of narrowing of the tracheal lumen due to granulation tissue have been described in about 18–24% of canines [32]. Severe lesions require intraluminal stent placement or surgical resection and end-to-end anastomosis [32]. Due to high costs of such treatment, the owner opted for euthanasia.

The presented case report has a few limitations. It is a single case report and not a case control study which affects the validity of our hypothesis. Nevertheless, we suspect, that manual TPE improved the neurological status of the dog, as it started to constantly improve the day after initiated treatment. However, it is unknown, whether manual TPE resulted in the dog’s clinical improvement or if similar clinical outcome would have been seen with ongoing supportive care only.

Unfortunately there is no commercial test available to measure serum anti-ganglioside Abs concentration in dogs with ACP thus a comparison between Abs titer before and after treatment was not possible. We can only state, that manual TPE procedure performed in this case report was safe for the patient. Another limitation was the single treatment with TPE. Consecutive treatments should be performed at 24–48 h intervals for greater reduction of antibody load. Two or three TPE procedures are routinely used in dogs with other immune-mediated diseases like IMHA, ITP and myasthenia gravis [6, 24, 25]. Using a single procedure might remove intravascular antibodies but without influence on the extravascular distribution. Furthermore, a rebound from ongoing antibody production and redistribution from the extravascular to intravascular space might occur after a single treatment with TPE instead of using multiple procedures. In this dog, the single TPE procedure was performed, because he started to improve after 24 h of the treatment. The improvement was seen every day that allowed him to be weaned off the mechanical ventilator on day 3 and return to neurological department on day 5. Another reason for single TPE procedure was the financial limitation of the owners.

In summary, the use of TPE for the treatment of ACP in the presented case was well tolerated. This is the first case report presenting manual TPE in a dog with presumed acute canine polyradiculoneuritis. The goal of a single manual TPE to rapidly improve the neurological condition of the dog was achieved without significant complication. Due to complications of the tracheostomy and euthanasia of the dog, the effectiveness of the treatment with respect to the recovery time could not be evaluated, but it can be assumed that the therapy contributed to faster wean off from MV. This case report confirms that TPE is a safe method and should be considered as an additional therapy in dogs with ACP especially with more severe signs of life threating paralysis of the respiratory muscles. A larger prospective study is warranted to assess the effectiveness of TPE in the treatment of ACP.

## Electronic supplementary material

Below is the link to the electronic supplementary material.

Supplementary Material 1

## Data Availability

All data generated or analysed during this study are included in this published article.

## Declarations

### Prior publication

Data have not been published previously.

## Abbreviations

AIHA: immune-mediated haemolytic anaemia
ACP: acute canine polyradiculoneuritis
CMAP: compound action potentials
GBS: Guillain–Barré Syndrome
IVIg: intravenous immunoglobulin
ITP: immune-mediated thrombocytopenia purpura
MMVD: Myxomatous mitral valve degeneration
MNCV: motor neve conduction velocity
MV: mechanically ventilated
RNS: repetitive nerve stimulation
TPE: therapeutic plasma exchange

## References

[1] Dewey CW, Da Costa RC (2016). Practical guide to canine and feline neurology.

[2] De Lahunta A, Glass E, Kent M (2015). Veterinary neuroanatomy and clinical neurology.

[3] Rupp A, Galban-Horcajo F, Bianchi E, Dondi M, Penderis J, Cappell J (2013). Anti-GM2 ganglioside antibodies are a biomarker for acute canine poliradiculoneuritis. J Peripher Nerv Syst.

[4] Anor S (2014). Acute lower motor neuron tetraparesis. Vet Clin North Am Small Anim Pract.

[5] Hirschvogel K, Jurina K, Steinberg TA, Matiasek LA, Matiasek K, Beltran E (2012). Clinical course of acute canine polyradiculoneuritis following treatment with human IV immunoglobulin. J Am Animal Hosp Assoc.

[6] Vitalo A, Buckley G, Londono L (2021). Therapeutic plasma exchange as adjunct therapy in 3 dogs with myasthenia gravis and myasthenia-like syndrome. J Vet Emerg Crit Care.

[7] Hughes RAC, Cornblath DR (2005). Guillain- Barré syndrome. Lancet.

[8] Cuddon PA. Electrodiagnosis in veterinary neurology: Electromyography,Nerve conduction studies and evoked potentials

[9] Ipe TS, Davis AR, Raval JS (2021). Therapeutic plasma exchange in myasthenia gravis: a systemic literature review and meta-analysis of comparative evidence. Front Neurol.

[10] Hofbauer N, Windberger U, Schwendenwein I, Tichy A, Eberspächer E (2016). Evaluation of canine red blood cell quality after processing with an automated cell salvage device. J Vet Emerg Crit Care.

[11] Kopecny L, Palm CA, Naylor S, Kirby J, Cowgill LD (2020). Application of therapeutic plasma exchange in dogs with immune-mediated thrombocytopenia. J Vet Intern Med.

[12] Rosenthal MG, Labato MA (2019). Use of therapeutic plasma exchange to treat nonsteroidal anti-inflammatory drug overdose in dogs. J Vet Intern Med.

[13] Francey T, Schweighauser A (2019). Membrane-based therapeutic plasma exchange in dogs: prescription, anticoagulation, and metabolic response. J Vet Intern Med.

[14] Ward DM (2011). Conventional apheresis therapies: a review. J Clin Apher.

[15] Kaplan AA (2013). Therapeutic plasma exchange: a technical and operational review. J Clin Apher.

[16] Buzzigoli SM, Genovesi M, Lambelet P, Logi C, Raffaelli S, Cattano D (2010). Plasmapheresis treatment in Guillain-Barré Syndrome: potential benefit over intravenous immunoglobulin. Anaesth Intensive Care.

[17] Salas-Ortiz P, Velez-Van-Meerbeke A, Galvis-Gomez CA, Rodriguez JH (2016). Human immunoglobulin versus plasmapheresis in Guillain-Barré Syndrome and Myasthenia gravis: a meta-analysis. J Clin Neuromuscul Dis.

[18] van der Meché FGA, Schmitz PIM (1992). A randomized trial comparing intravenous immune globulin and plasma exchange in Guillain-Barré syndrome. N Engl J Med.

[19] Bril V, Ilse WK, Pearce R, Dhanani A, Sutton D, Kong K (1996). Pilot trial of immunoglobulin versus plasma exchange in patients with Guillain-Barré syndrome. Neurology.

[20] Randomized trial of (1997). Plasma exchange, intravenous immunoglobulin, and combined treatments in Guillain-Barré syndrome. Plasma Exchange/Sandoglobulin Guillain-Barré Syndrome Trial Group. The Lancet.

[21] Diener H-C, Haupt WF, Kloss TM, Rosenow F, Philipp T, Koeppen S (2001). A preliminary, randomized, multicenter study comparing intravenous immunoglobulin, plasma exchange, and immune adsorption in Guillain-Barré syndrome. Eur Neurol.

[22] Stanciu GD, Musteata M, Armasu M, Saftencu PM, Solcan G (2014). Electrophysiological aspects in idiopathic acute canine polyradiculoneuritis. Bull UASVM Veterinary Med.

[23] Vandevelde M, Higgins JR, Oevermann A. Veterinary neuropathology. Essentials of theory and practice. Wiley Blackwell; 2012. p. 78.

[24] Francey T, Etter M, Schweighauser A (2021). Evaluation of membrane-based therapeutic plasma exchange as adjunctive treatment for immune‐mediated hematologic disorders in dogs. J Vet Intern Med.

[25] Kopecny L, Palm CA, Naylor S, Kirny J, Cowgill LD (2020). Application of therapeutic plasma exchange in dogs with immune-mediated thrombocytopenia. J Vet Intern Med.

[26] Culler CA, Reinhardt A, Vigani A (2020). Successful management of clinical signs associated with hepatic encephalopathy with manual therapeutic plasma exchange in a dog. J Vet Emerg Crit Care.

[27] Shang P, Zhu M, Baker M, Feng J, Zhou Ch, Hong-Liang Z (2020). Mechanical ventilation in Guillain- Barré syndrome. Expert Rev Clin Immunol.

[28] Berg B, Storm EF, Garssen MJP, Blomkwist-Markens PH, Jacobs BC (2018). Clinical outcome of Guillain- Barré syndrome after prolonged mechanical ventilation. J Neurol Neurosurg Psychiatry.

[29] Kalita J, Ranjan A, Misra UK (2016). Outcome of Guillain- Barré syndrome patients with respiratory paralysis. QJM.

[30] Rutter ChR, Rozanski EA, Sharp CR, Powell LL, Kent M (2011). Outcome and medical management in dogs with lower motor neuron disease undergoing mechanical ventilation: 14 cases (2003–2009). J Vet Emerg Crit Care.

[31] Regan K, Hunt K (2008). Tracheostomy management. Continuing Educ Anaesth Crit Care Pain.

[32] Johnson SA, Tobias KM. Veterinary surgery small animal. 2nd ed. Elsevier; 2018. 4574 – 5.25.

