# Supplementary material for: Manual therapeutic plasma exchange for treatment of a dog with suspected acute canine polyradiculoneuritis
Source: Acta Vet Scand. 2023 Mar 27;65:14. doi: 10.1186/s13028-023-00675-0 (PMC10044371; doi:10.1186/s13028-023-00675-0)
Supplement: Supplementary file 1 — Supplementary Material 1 [file 13028_2023_675_MOESM1_ESM.docx]

Additional file 1

| **Day of hospitalization** | **1** | **2** | **3** | **4** | **5** | **6a** | **6b** | **7a** | **7b** |
| --- | --- | --- | --- | --- | --- | --- | --- | --- | --- |
| pH  (7.33 – 7.43) | 7.45 | 7.34 | 7.43 | 7.41 | 7.36 | 7.41 | 7.34 | 7.49 | 7.40 |
| pCO2 [mmHg]  (30.0 – 47.7) | 34.4 | 49.5 | 41.5 | 45.1 | 55.0 | 61.0 | 69.9 | 46.6 | 53.6 |
| HCT [%]  (36.9 – 52.4) | 52.0 | 48.1 | 36.3 | 37.4 | 40.0 | 27.4 | 23.2 | 19.3 | 26.7 |
| Na [mmol/L]  (145.8 – 151.8) | 144.0 | 147.2 | 141.0 | 140.8 | 145.3 | 145.8 | 144.1 | 145.3 | 147.0 |
| K [mmol/L]  (3.56 – 4.80) | 4.00 | 4.10 | 4.25 | 4.12 | 3.77 | 3.12 | 2.84 | 3.34 | 4.11 |

**1.** 05.11.20 13:52 (Day of admission); **2.** 06.11.2020 18:58 (Beginning of mechanical ventilation); **3.** 07.11.20 16:04 (arterial); **4.** 08.11.20 09:29; **5.** 09.11.20 09:09; **6a.** 10.11.20 17:13 (during plasmaphoresis, Ca-substitution (1 ml/kg) thereafter); **6b.** 10.11.20 18:40 (immediately after plasmaphoresis, Ca-Substitution (1 ml/kg) thereafter); **7a.** 11.11.20 04:50; **7b.** 11.11.20 19:00
